# Microbial Safety and Sensory Analyses of Cold-Smoked Salmon Produced with Sodium-Reduced Mineral Salts and Organic Acid Salts

**DOI:** 10.3390/foods11101483

**Published:** 2022-05-19

**Authors:** Even Heir, Maria Jacobsen, Mari Øvrum Gaarder, Ingunn Berget, Paw Dalgaard, Merete Rusås Jensen, Askild L. Holck

**Affiliations:** 1 Nofima AS—Norwegian Institute of Food, Fisheries and Aquaculture Research, P.O. Box 210, N-1431 Ås, Norway; mariajacobsen150@hotmail.com (M.J.); mari.gaarder@nofima.no (M.Ø.G.); ingunn.berget@nofima.no (I.B.); merete.rusas.jensen@nofima.no (M.R.J.); askild.holck@nofima.no (A.L.H.); 2 National Food Institute (DTU Food), Technical University of Denmark, Kemitorvet, Bldg 202, DK-2800 Kongens Lyngby, Denmark; pada@food.dtu.dk

**Keywords:** cold-smoked salmon, *Listeria monocytogenes*, sodium reduction, organic acid salts, sensory profile, potassium chloride, food safety

## Abstract

Cold-smoked (CS) salmon contains high levels of sodium salts, and excess dietary sodium intake is associated with an array of health complications. CS salmon may also represent a food safety risk due to possible presence and growth of the foodborne pathogen *Listeria monocytogenes* which may cause fatal human infections. Here we determine how reformulated CS salmon using commercial sodium-reduced salt replacers containing KCl (e.g., Nutek, Smart Salt, SOLO-LITE) and acetate-based preservative salts (Provian K, proviant NDV) affect sensory properties, quality, and microbial safety. Initial sensory screening of sodium-reduced CS salmon was followed by *L. monocytogenes* growth analyses in selected variants of reformulated CS salmon, and finally by analyses of CS salmon variants produced in an industrial smokehouse. Projective mapping indicated overall minor sensory changes in sodium-replaced samples compared with a conventional product with NaCl. Growth of *L. monocytogenes* was temperature-dependent (4 °C vs. 8 °C storage) with similar growth in sodium-reduced and conventional CS salmon. The addition of 0.9% of the preservative salts Provian K or Provian NDV gave up to 4 log lower *L. monocytogenes* counts in both sodium-reduced and conventional cold-smoked salmon after 29 days of chilled storage. No changes in pH (range 6.20–6.33), a_w_ levels (range 0.960–0.973), or weight yield (96.8 ± 0.2%) were evident in CS salmon with salt replacers or Provian preservative salts. Analyses of CS salmon produced with selected mineral salt and preservative salt combinations in an industrial salmon smokery indicated marginal differences in sensory properties. Samples with the preservative salt Provian NDV provided *L. monocytogenes* growth inhibition and low-level total viable counts (<2.8 log/g) dominated by *Photobacterium* and *Carnobacterium* during storage. Production of sodium-reduced CS salmon with inhibiting salts provides a simple method to achieve a healthier food product with increased food safety.

## 1. Introduction

Excess dietary sodium intake increases the risks for development of serious health complications. This includes hypertension (high blood pressure), which is strongly associated with cardiovascular diseases and premature deaths [1,2,3]. High sodium intake is therefore among the most serious health challenges worldwide. In many countries, the average daily consumptions of salt (as NaCl) are in the range of 8–12 g, around twice the recommended maximum daily intake level of 5 g, equivalent to 2 g sodium per day [4,5,6]. Consumption of foods processed by industry or prepared in restaurants provides approximately 75% of the sodium intake [7]. National policies on salt reduction implemented in many European countries have indicated that combinations of several measures (e.g., salt targets of food, mandatory labelling of high-salt food and consumer education) have reduced population salt intake [8]. In Norway, a national salt partnership exists to stimulate actions on salt reduction in food products and served meals. This organization has established indicative salt targets for about 100 products. The salt target for cold-smoked fish in the period 2019–2021 is set to 3.0 g sodium chloride/100 g product, corresponding to 1.2 g sodium. This salt target is consistent with the criteria for use of the Nordic Keyhole label on food products aimed to provide an easier choice of healthier foods among consumers [9]. Salt levels in cold-smoked (CS) salmon are typically in the range 2–4% [10,11,12], but further sodium reductions will be a goal according to health, consumer demand, and labelling issues. Present and future salt targets require evaluations and knowledge on strategies for sodium-reduced CS salmon without compromising processability, sensory properties, shelf life, and food safety.

Smoked salmon is not among the major contributors to dietary sodium. However, it is an industrially processed product distributed and consumed worldwide with significant economic impact and with variants having higher salt levels than needed. Another risk factor of smoked salmon products is the foodborne pathogen *L. monocytogenes* causing human listeriosis [13,14]. Although human listeriosis has a low incidence, this potential serious infection with fatality rates of 20–30% has placed *L. monocytogenes* among the top five pathogens responsible for the greatest burden of costs of illness and loss of quality-adjusted life years (QALY; [15,16]). It is also a most challenging and economically costly bacteria for many salmon processors due to their production of ready-to-eat salmon products suffering from frequent product contaminations followed by product recalls and extensive *Listeria* control and testing programs. To reduce the health burden, improve competitiveness, and meet the demands of consumers and authorities for healthy, safe CS salmon products with reduced salt, there is a need for the salmon processing industry to reformulate CS salmon products to reduce the risks associated with both sodium content and the microbial food safety (mainly *L. monocytogenes*) of such products. 

An obvious impact of salt in CS salmon is the provided salty taste, but it is also needed for the functionality, processability, microbial stability, and shelf life of the final product. Salt types and levels affect proteins, lipids, and biochemical processes in the salmon. This can further influence water binding in the product and thereby yield, texture, sensory properties, and water activity affecting growth of both microbial spoilers and pathogens [11,17,18]. A certain level of added salts is therefore needed to obtain characteristic CS salmon products. Strategies for sodium reduction must maintain the processing and product properties provided by sodium. Among the variety of salt substitutes including yeast extracts, herbs, and spices, exclusively mineral salts are regarded to provide CS salmon with the needed multi-functional properties related to processability and quality parameters, including texture and water-holding capacities, sensory properties, microbial safety, and shelf life [19,20]. Though, sodium-reduced mineral salts such as potassium, calcium, and magnesium salts may stimulate perceptions including bitter, salty, metallic, sour, and astringent off-flavors [21,22].

Potassium chloride (KCl) and sodium chloride (NaCl) share several key technological properties, and KCl-based salts are therefore a most promising sodium chloride salt substitute. Still, effects of potassium on specific health, food quality, safety, and sensory issues need to be clarified to utilize the potential for production of sodium reduced CS salmon. Although reduced and increased dietary intakes of sodium and potassium, respectively, have health beneficial effects on a population basis, adverse health effects of excessive intake of potassium have also been reported in vulnerable groups including patients with renal failures, heart failures, and diabetes mellitus ([23] and references therein). A risk–benefit analysis in United Kingdom concluded that the benefits outweighed the potential risks at a population level if 15–25% of dietary sodium was replaced with potassium [24]. Therefore, smoked salmon as a significant contributor to excess potassium intake seems limited. Still, the conclusions with respect to the risks of KCl as a salt substitute remain ambiguous and may restrict the willingness of CS salmon manufacturers to implement KCl as a salt substitute in their production. Processors of CS salmon may also resist to replace NaCl with salt substitutes in a successful product with long tradition without thorough evaluation and documentation on how this will affect key quality and food safety product characteristics and parameters.

Boziaris et al. reported that partial substitution of NaCl with equimolar levels of KCl does not affect bacterial *Listeria* growth in cold-smoked salmon [25]. This means that in practice, *Listeria* food safety challenges are similar, though often significant, in products with either type of these mineral salts at levels typical for CS salmon. Strategies to improve the food safety and health effects of CS salmon through salt reduction should therefore also include strategies to reduce the microbial risks of such products. Application of salts of organic acids (e.g., Provian K) also including “label-friendly” fermentates (e.g., Provian NDV) is a strategy indicated to inhibit growth of *L. monocytogenes* in conventional (containing NaCl) CS salmon while maintaining the organoleptic properties of the product [26]. This also suggests that such compounds may contribute to sodium-reduced CS salmon with acceptable or enhanced sensory characteristics and improved food safety.

The aim of the current study was to determine how sodium-reduced salt substitutes affected sensory properties, microbial safety, and quality of CS salmon. The study comprised a sensory screening of CS salmon produced with commercial sodium-reduced KCl containing mineral salts followed by production of CS salmon with KCl-based mineral salts and sodium-free acetate-based preservative salts to evaluate effects on *L. monocytogenes* growth. Finally, CS salmon was produced with combinations of selected mineral and preservative salts in an industry cold-smoked salmon processing plant and subjected to fully descriptive sensory and microbial analyses.

## 2. Materials and Methods

### 2.1. Mineral Salts and Organic Acid Salts 

Commercially available KCl-based mineral salts (*n* = 7) were obtained from manufacturers (Table 1, Treatments no. 3–9). Sodium chloride and KCl were included as control mineral salts. The organic acid salts Provian K (blend of potassium acetate and potassium diacetate) and “label-friendly” Provian NDV (buffered dry vinegar potassium salt made from naturally fermented vinegar) were obtained from Niacet b.v. (Tiel, The Netherlands). The salts were used as ingredients in the dry salting procedure of fresh salmon fillets prior to cold-smoking as described below.

### 2.2. Production of Cold-Smoked Salmon with Salt Replacers

#### 2.2.1. Salmon Produced for Screening the Sensory Effects of Salt Replacers

Fillets of fresh, skin-on, ice-stored Norwegian, farmed Atlantic salmon (*Salmo salar*) were received from Lerøy Seafood Group ASA. The fillets were cut to obtain the Norwegian and Scottish Quality Cut (NQC, SQC) [27]. Salts and salt mixtures of NaCl, KCl, and commercial sodium-reduced salt replacers were prepared as described below (2.2.3). Fillets were dry-salted with salt levels according to the weight of each salmon fillet (Table 1, Treatment no. 1–9). Prior to salting, the fillets were placed in separate plastic bags to avoid spill and obtain controlled salt levels. The plastic bags with salted fillets were sealed under mild vacuum and stored at 4 °C for 64–68 h for even distribution of salts in the fillets. After salt distribution, the salmon fillets were unpacked and weight yields after salting were determined. The salmon fillets were cold-smoked in a programmable smoking cabinet (DOLESCHAL, process control unit SC2000; Inject Star Maschinenbau GmbH, Hagenbrunn bei Wien, Austria) using smoke generated from beech chipwood (Räuchergold KL 2/16; J. Rettenmaier & Söhne GmbH, Rosenberg, Germany).

The fillets were placed horizontally on stainless steel meshes and cold-smoked. The cold-smoking was performed at 25 °C and included an initial drying step of 30 min with air circulation, followed by five cycles of smoking/smoke circulation for a total of approximately 3.5 h. Smoking was followed by weight yield determination, vacuum packing, and storage of each fillet at 0 °C for approximately 64 h to allow time for diffusion of smoke compounds in the salmon fillets prior to the *L. monocytogenes* contamination experiments. The vacuum-packed, smoked fillets were stored frozen (−20 °C) until sensory analyses. 

#### 2.2.2. Salmon Produced for *L. monocytogenes* Challenge Tests

Mineral salts and blends of mineral salts and organic acid-based preservative salts were used to produce CS salmon applied in *L. monocytogenes* challenge tests. In total, 16 different combinations of salt mixtures of mineral salts and preservative organic acid-based salts were used (Table 1, products 1–4 and 10–21). The mineral salts were selected to include NaCl (control), an in-lab prepared sodium-reduced salt mixture of NaCl (80%) and KCl (20%), and the sodium-reduced salt replacers Smart Salt 40 and NuTek 78300. The latter two represented commercial salt replacers regarded as suitable for use in CS salmon according to levels of KCl (20–30%) and sensory screening results. They also represented salts where NaCl was partially substituted by KCl but with Smart Salt 40 also having MgCl_2_ as a significant mineral salt ingredient (Table 1). The preservative organic acid-based salts Provian K (P-K) and Provian NDV (P-NDV) were included at two levels (0.5% and 0.9%) in salt mixtures used for production of CS salmon with NaCl (control) or sodium-reduced (blend of NaCl and KCl) at two total mineral salt levels (2.5% and 3.0%). The salting, cold-smoking, and packaging of the salmon fillets were as described above (Section 2.2.1). The CS salmon were stored vacuum-packed at −40 °C prior to the challenge tests.

#### 2.2.3. Industrially Produced Salmon

A subset of the different salt mixtures was selected for production and subsequent sensory, microbial safety, and quality evaluation of CS salmon (Product P1–P6; Table 2) produced in an industrial CS salmon processing plant. The salt mixtures were selected to compare and determine the effects of sodium-reduced salt replacers with and without added preservative salt (P-NDV) to conventional CS salmon produced with NaCl. Prior to dry-salting, salt mixtures were prepared to obtain 30% replacement of NaCl with KCl in the sodium-reduced mixtures. All salt mixtures had added sucrose to a final level of 25% according to presence of sucrose in the conventional NaCl/sucrose mix (product P6) applied by the producer. Salt mixtures of P4 and P5 were added to Provian NDV. The salt mixes of P1-P5 were applied in excess according to the manufacturer’s experience and conventional salting protocol to obtain appropriate levels (approximately 3%) of salt in the salmon fillets. The salmon fillets were exposed to the salt mixes for 24 h 4 °C, rinsed in fresh water, dried to remove excess water, and smoked using the in-house protocol of the manufacturer. Product P6 was CS salmon produced following the manufacturer’s standard, in-house automated salting and smoking protocol. CS salmon fillets were individually vacuum-packed and stored at 4 °C. Microbial analyses were performed on days 6, 14, and 34 after smoking.

### 2.3. Sensory Analyses

All sensory analyses performed in the present experiment were carried out using a highly trained sensory panel. The trained panel consisted of 10 professional assessors employed at Nofima (Ås, Norway). They are regularly tested and trained according to ISO 8586:2012, and the sensory laboratory follows the practice of ISO 8589:2007. 

Preparation of all samples was similar regardless of sensory method. They were all taken from the dorsal part of the quality cuts (SQC & NQC), and excess fat was removed. Each assessor received two slices, approximately 3 mm thick and vertically cut. Each sample was served at room temperature (18 ± 2 °C) in separate odorless plastic containers labelled with three-digit random numbers. Water and crackers were available for palatal rinsing between samples.

#### 2.3.1. Sensory Screening by Projective Mapping of CS Salmon with Salt Replacers

CS salmon produced with standard NaCl (Product no. 1, two replicate samples; Table 1) or sodium salt substitutes (Products no. 2 through 9) were evaluated for the sensory modality taste by Nofima’s trained panel using projective mapping (PM) [28,29]. The assessors were asked to taste and evaluate each sample in random order and place them according to similarities or dissimilarities with respect to taste. After completing the task, they were asked to provide a written description of each of the samples. The whole session took place individually and was registered in the program EyeQuestion^®^, Logic8 BV, Utrecht, The Netherlands.

#### 2.3.2. Sensory Descriptive Analyses of Industrially Produced CS Salmon with Salt Replacers

A full sensory descriptive analysis (DA) according to the “Generic Descriptive Analysis” described by Lawless and Heymann [30] and the ISO standard 13299:2016 was performed on the industrially produced CS salmon (P1-P6, Table 2) stored for 14 days at 4 °C after smoking. The trained assessors (*n* = 8) were instructed to evaluate the intensity of 23 sensory attributes related to smell, taste, and texture (Table S1).

The intensity was evaluated for all samples on an unstructured scale (15 cm line scale) for each attribute. Selection and description of the attributes were based on results from the PM, and by the assessors in a pre-trial with two different samples (Control and Nutek 0.9% Provian NDV) guided by the panel leader. Each different salt treatment (P1-P6) was randomly evaluated on six individual CS salmon fillets, resulting in a total of 36 samples served in nine sessions. The samples were served in blind trials randomized according to sample, assessor, and replicate. The evaluation took place individually and results were registered in the software EyeQuestion^®^. The software transformed the responses into numbers between 1 (low intensity) and 9 (high intensity).

**Table 2:** Characteristics of industrially produced CS salmon.

Product No.	Salt Mixture ^1^	Added NaCl, KCl, Total Mineral Salts (%) ^2^			Added Provian-NDV salt (%) ^2^	Measured Product Characteristics and Salt Uptake (%) ^3^					
		NaCl	KCl	Total		NaCl	% NaCl Uptake	KCl	% KCl Uptake	Acetate	% Acetate Uptake ^3,4^
P1	NaCl	4.13	0	4.13	0	1.89	46	ND	ND	0.01 ± 0.002	ND
P2	NaCl + KCl	2.89	1.24	4.13	0	1.46	51	0.62 ± 0.03	50	ND	ND
P3	Nutek 78300	2.89	1.24	4.13	0	1.86	64	0.68 ± 0.07	55	ND	ND
P4	NaCl + P-NDV	4.13	0	4.13	1.24	1.51	37	ND	ND	0.17 ± 0.1	23
P5	Nutek 78300 + P-NDV	2.89	1.24	4.13	1.24	1.65	57	0.68 ^5^	55	0.23 ± 0.1	31
P6	Standard product ^6^	ND	0	ND	0	2.48	ND	ND	ND	ND	ND

^1^ P-NDV: Provian NDV; ^2^ Salt levels in weight % of the fresh fish, ND: Not determined. ^3^ Calculated levels of NaCl and KCl are according to measured total levels of Na and K (subtracted the background levels of Na^+^ (59 mg/100 g) and K^+^ (363 mg/100 g) in farmed Atlantic salmon) [31]. Salt uptake is the level of absorbed salt in CS salmon relative to the added amount of the respective salts. ^4^ Levels were calculated according to added levels of Provian-NDV containing 100 % K-acetate. ^5^ Level of KCl is provided by added Nutek and assumed to be the same as in Product no. P3. ^6^ Commercial CS salmon produced using NaCl and the traditional in-house dry salting and smokery process.

### 2.4. Listeria monocytogenes Challenge Tests

#### 2.4.1. *L. monocytogenes* Strains and Culture Conditions

*L. monocytogenes* strains used in the experiments are displayed in Table 3. The ten strains used included six strains isolated from salmon and salmon processing facilities, three strains associated with human listeriosis outbreaks, and one strain from cattle. The strains represented three serovars commonly associated with human listeriosis and various multilocus sequencing types (ST). The strains were maintained at −80 °C in Brain Heart Infusion (BHI) broth with 15% glycerol. Inocula for the challenge tests on CS salmon were prepared from single colonies of each strain grown on BHI agar (30 °C, 24 h) and inoculated into 3 mL BHI broth before incubation (30 °C for 48 h). This pre-culture was used for inoculation (1%) of each strain in individual tubes of 3-mL BHI broth. After incubation at 30 °C for 24 h, the bacterial cultures were mixed to contain equal cell numbers of each strain. The 10-strain cell culture mix was stored at 4 °C for 20–24 h for cold adaptation. Dilutions to working solutions (5 × 10^4^ cfu/mL) were performed in 0.9% NaCl.

#### 2.4.2. *L. monocytogenes* Contamination and Growth in CS Salmon

CS salmon fillets were thawed overnight at 4 °C. A contamination scenario reflecting contamination with *L. monocytogenes* during the slicing process was used. The CS salmon fillets were sliced, and slices of approximately 5 g were each treated with 20 µL of the 10-strain *L. monocytogenes* cocktail (5 × 10^4^ cfu/mL) on the surface before non-inoculated 5 g slices of salmon were placed on the *L. monocytogenes*-contaminated salmon surface to obtain 10 g samples. The 20 µL *L. monocytogenes* cocktail was spread on the surface by using a sterile bacterial cell spreader. The samples were placed into separate stomacher bags, vacuum-packed, and stored at either 4 °C or 8 °C. Non-inoculated samples of CS salmon were stored under the same conditions and were used to determine total counts and indigenous background microbiota. The challenge test and analyses were repeated on a separate day using CS salmon from the same production. All experiments with *L. monocytogenes* were performed in a biosafety level 3 pilot processing plant.

### 2.5. Microbiological Analyses and Bacterial Identification

Bacterial counts in the CS salmon fillets were determined after 0, 7, 12, 19, and 29 days after start of storage at 4 °C and 8 °C if not otherwise stated. At each sampling day, two to four parallels of each of the sixteen CS salmon types produced (Section 2.2.2.) were analysed. To each sample in stomacher bags, 40 mL peptone water was added. The samples were stomached for 60 s and appropriate 10-fold dilutions in peptone water were plated on Rapid L’mono agar (Bio-Rad, France) and incubated at 37 °C for 24 h for *L. monocytogenes* quantification. Total viable counts were determined by plating on blood agar plates (Blood agar base, 5% defibrinated horse blood, Thermo Fisher Scientific, Oslo, Norway) and aerobic incubation at 15 °C for five days. Two to four replicate samples were analyzed at each sampling point.

Identification of bacteria representing the overall microbiota of industrially produced CS salmon was determined by MALDI-TOF MS (Bruker Daltonic, GmbH, Bremen, Germany). Analyzed salmon samples (P1, P3, P4, P6) included products containing NaCl (P1, P6), the sodium-reduced salt replacer Nutek 78300 (P3), and a blend of NaCl and the preservative salt P-NDV (P4). At the end of storage, 30 blood agar-grown colonies from each CS salmon sample were randomly picked and grown to pure culture before small portions of the individual colonies were collected by a sterile toothpick and smeared onto a MBT Biotarget 96 plate (Bruker Daltonic). The sample spot was treated with 1 µL of HCCA matrix (α-cyano-4-hydroxycionnamic acid) and air-dried.

Mass spectra were obtained using a Microflex^TM^ LT, the manufacturer’s standard operating procedure (Revision 3; August 2013) and flexControl 3.4 software (Bruker Daltonic). Bacterial identification was determined by comparing mass spectra of the analyzed samples to the internal Bruker database (MBT Compass 4.1) in addition to a self-constructed database generated from the in-house strain collection of food-borne bacteria already identified by 16S rRNA gene sequencing. Accuracy of identification was assured by obtained score values. Bacterial samples with score values below the threshold value of 1.7 resulting in no bacterial identification were identified by partial 16S rRNA gene sequencing according to a previously reported protocol [36].

### 2.6. Physiochemical Analyses

Levels of Cl^−^ were determined by an automated potentiometric titration method using 0.1 M AgNO_3_ [37]. Quantitative levels of organic acids in cold-smoked salmon (Treatment no. 10 through 21; Table 1) were determined by high-pressure liquid chromatography [38]. External standards of lactic acid (Sigma L1750) and acetic acid (Merck, LiChropur^®^) were used for identification and quantification of the compounds. Samples (5 g) were analyzed in duplicate. Sodium and potassium were determined in duplicate by inductively coupled plasma–mass spectrometry (ICP-MS) according to the reference methods SS-EN ISO 17294-2:2016/SS-EN 13805:2014. The pH of CS salmon samples was measured in the stomaching solution of a 5 g sample in 25 mL deionized water using a sensION + pH 31 pH meter (Hach Company, Loveland, CO, USA). The smoke component phenol was estimated by a spectrophotometric method [39]. Water activity (a_w_) of CS salmon was measured at room temperature (Aqualab, series 3TE, Decagon Devices Inc., Washington, USA). Weight yields (%) were determined by weighing the fillets before salting, prior to smoking, and after smoking, and yields were calculated relative to the weight of the raw fillets. 

### 2.7. Experimental Design and Statistical Analyses

The PM sensory data was collected as X and Y coordinates of each sample on each assessor’s individual map. A multiple factor analysis (MFA) was performed considering the X and Y coordinates for each sample per assessor map [29,40]. The PM sensory analyses were done with the software XLstat, 2021, version 4.1, addinsoft, New York.

The descriptive sensory data were analysed with analysis of variance (ANOVA) using a linear mixed model comprising the factors: product (P1–P6, Table 2), assessors, replica, and the second-order interactions. Assessors and interactions involving assessors were considered random, whereas the other factors were fixed. Mean intensities were calculated, and significant differences were checked using TUKEY’s HSD test (*p* < 0.05). Principal component analysis (PCA) was performed to visualize and explore effects of different salt replacers. The descriptive sensory analyses were done with EyeOpenR in the software EyeQuestion.

Growth of *L. monocytogenes* was modelled using analysis of variance (ANOVA) and log (base 10) of the bacterial count as the dependent variable. Five different analyses (Table S2) were performed to investigate the following experimental factors on *L. monocytogenes* growth in the different CS salmon products (Table 1). 

(i) Effect of different mineral salts (Products no. 1-4); (ii) Effect of Provian K (0, 0.5%, and 0.9%) and salt (NaCl or sodium-reduced) (Products no. 10-13); (iii) Effect of Provian NDV (0, 0.5% and 0.9%) and salt (NaCl or sodium-reduced) (Products no. 16-19); (iv) Comparison of Provian type (K or NDV), concentration (0.5% or 0.9%), and salt (NaCl or sodium-reduced; Products no. 10-13 vs. 16-19); (v) Effect of reduced levels of total mineral salts (2.5%) combined with different Provian types and concentrations (Products no. 14–15 and 20–21).

In all models, the experimental factors (as listed above), days of storage (7, 12, 19, 29), and temperature (4 or 8 °C) were included in the model, allowing for main effects and up to three-way interactions. Analysis of variance (ANOVA) was used to determine statistically significant effects on the bacterial levels by the treatments. Model reduction using backward elimination and the AIC (Akaike’s information criterion) was applied using the r-function step [41,42]. Linear contrasts were applied to compare treatments/factor levels and a significance level of α = 0.05 was used, meaning that the contrasts were considered statistically different for *p*-values < 0.05. All analyses were performed in R [43].

Total viable counts were analysed with one-way ANOVA for each day, including products P1-P5, a post-hoc comparison comparing each treatment against P1 was done using Dunnet’s test. P6 was compared to P1 in a separate analysis as the commercial samples varied much more than the test samples, and assumptions for ANOVA could not be met.

## 3. Results

An overview of the different CS salmon types produced for sensory analysis and *L. monocytogenes* challenge tests is presented in Table 1. The composition of the different mineral salts, the amount of added preservative organic acid salts, and the measured levels and calculated uptake of mineral salts (NaCl, KCl) and acetate in addition to phenol content are displayed.

### 3.1. Sensory Screening of CS Salmon with Sodium Reduced Salt Replacers

**Figure 1:**
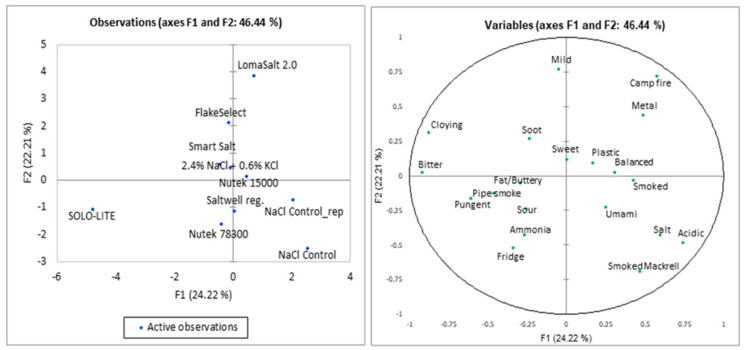
Sensory analysis by projective mapping. Observation plot and variables plot indicating terms mentioned six times or more, obtained from PM of 10 samples of CS salmon conducted by 10 trained assessors.

Projective mapping (PM) revealed a relatively low explained variance on the first two factors (F1 and F2; 46.44%) although the replicates (Control and Control-rep) were located closely together (Figure 1). This indicates generally small differences in sensory properties between the different products, indicating that many of the salt replacers have a potential for use in low-sodium CS salmon. CS salmon containing SOLO-LITE was perceived slightly more bitter and cloying, and with lower smoke taste than the other samples. Both products with SOLO-LITE and LomaSalt 2.0 were perceived as less salty and placed further away from the control in the two-dimensional observation plot. In addition, LomaSalt 2.0 was perceived as mild in taste.

### 3.2. Growth of L. monocytogenes in CS Salmon Produced with NaCl and Selected Salt Replacers 

**Figure 2:**
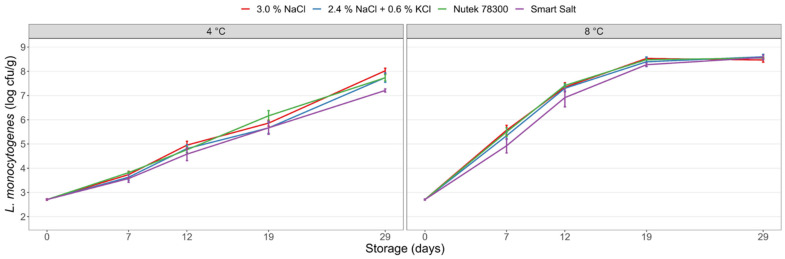
Growth of *L. monocytogenes* in CS salmon produced with NaCl (control) and different sodium-reduced mineral salts (Treatments no. 1-4; Table 1). The salmon fillets were treated with the different salts to a level of 3% (*w*/*w*). After smoking, the CS salmon was inoculated with *L. monocytogenes* at day 0 and stored under vacuum at 4 °C and 8 °C for 29 days. Mean values of two experiments (four to eight measurements per data point) and standard error of the mean are displayed.

The overall small differences obtained in the PM sensory screening allowed the selection of a subset of the mineral salts for further study in *L. monocytogenes* challenge tests. CS salmon produced with selected salt replacers demonstrated similar growth of *L. monocytogenes* as control CS salmon made with 3% NaCl during storage (Figure 2). *L. monocytogenes* grew to high levels (7–8 log cfu/g) within 29 days at 4 °C, while significantly higher *L. monocytogenes* levels (*p* < 0.001) were obtained at 8 °C storage, reaching maximum population density of approximately 8.2 log cfu/g after about 19 days of storage. ANOVA demonstrated a statistically significant main effect of salt type (*p* < 0.001) with CS salmon containing Smart Salt 40 (Treatment no. 4), having lower mean *L. monocytogenes* counts during storage. Though, the actual differences were small and were regarded to be of no biological relevance as the salt effect explained less than 1% of the variability in the data. (All ANOVA tables are provided in Supplementary Table S2). 

### 3.3. Effects of Organic Acid Based Preservative Salts on the Growth of L. monocytogenes in Sodium-Reduced CS Salmon

**Figure 3:**
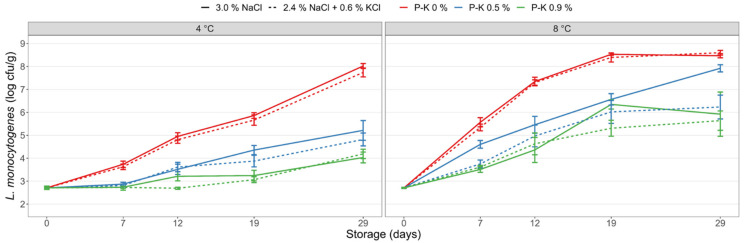
Growth of *L. monocytogenes* in CS salmon produced with 3% NaCl (control), a sodium-reduced reference salt (2.4% NaCl + 0.6% KCl) and with addition of the preservative salt Provian K. The Provian K salt was added at levels of 0.5% and 0.9% in the salting process. The CS salmon was inoculated with *L. monocytogenes* at Day 0 and stored under vacuum at 4 °C and 8 °C for 29 days. Mean values of two experiments (four to eight measurements per data point) and standard error of the mean are displayed.

Inclusion of the preservative salt Provian K in the dry salting process of CS salmon provided growth inhibition of *L. monocytogenes* during storage both in CS salmon produced with 3% NaCl and in the sodium-reduced samples (2.4% NaCl + 0.6% KCl; Figure 3). The *L. monocytogenes* levels were lower (*p* < 0.001) at all sampling days for CS salmon with Provian K compared with CS salmon without Provian K added. Provian K caused both increased lag times and reduced growth rates resulting in up to 4 log lower *L. monocytogenes* counts at end of storage for CS salmon with Provian K (treatments no. 10–13; Table 1) compared to salmon without Provian K (treatments no. 1 and 2; Figure 3). Growth inhibitory effects appeared dependent on the applied Provian K levels, though differences were not statistically significant at all time points. 

Overall, only minor differences in growth inhibitory effects in CS salmon containing the same level of Provian K were obtained when comparing control salmon (3% NaCl) with sodium-reduced salmon (2.4% NaCl + 0.6% KCl). Although statistically significant differences between control CS salmon and sodium-reduced salmon were evident for certain levels at certain storage temperatures and sampling days, these differences were regarded to be of minor biological relevance. At 4 °C storage, complete growth inhibition (<0.5 log increase) was obtained for sodium-substituted CS salmon with 0.9% Provian K stored for 19 days and with 1.5 log increase in *L. monocytogenes* during the 29-day storage period (Figure 3). CS salmon with no or 0.5% Provian K had a 5.0–5.3 or 2.1–2.5 log increase, respectively, during 29 days of storage. At 8 °C storage, growth inhibition of *L. monocytogenes* by the Provian K salt appeared less robust with *Listeria* levels increasing in the range 3.5–5.2 log and 2.9–3.2 log at Provian K salt levels of 0.5% and 0.9%, respectively. In CS samples without Provian K salts, *L. monocytogenes* increased with ≥5.7 log after 19 days of storage (Figure 3). 

**Figure 4:**
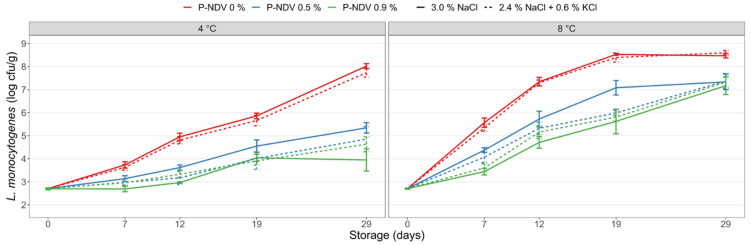
Growth of *L. monocytogenes* in CS salmon produced with 3% NaCl (control), a sodium-reduced reference salt (2.4% NaCl + 0.6% KCl), and with addition of the preservative salt Provian NDV. The Provian NDV salt was added at levels of 0.5% and 0.9% levels in the salting process. The CS salmon was inoculated with *L. monocytogenes* at Day 0 and stored under vacuum at 4 °C and 8 °C for 29 days. Mean values of two experiments (four to eight measurements per data point) and standard error of the mean are displayed.

CS salmon produced with the label-friendly fermentate Provian NDV gave results similar to those which had added Provian K (Figure 4). Also here, the levels of *L. monocytogenes* were lower in Provian NDV containing samples compared with controls at all timepoints (*p* < 0.001). Moreover, an overall dose response effect was seen at both 4 °C and 8 °C with higher inhibition obtained using increased Provian NDV levels. This effect was evident both for sodium-reduced samples and for samples with 3% NaCl (*p* ≤ 0.035). No effect of salt type was observed. Analyses demonstrated interaction effects between salt type and Provian NDV, but this interaction explained <1% of the variation in the data and thus has no practical significance. Factors influencing *L. monocytogenes* levels were mainly Provian NDV concentration (at 4 °C storage), storage temperature, and storage time (Supplementary Table S2). After 29 days of storage at 4 °C, mean levels of *L. monocytogenes* in CS salmon with 0.5% and 0.9% added Provian NDV were 2.8 log and 3.6 log lower, respectively, compared with CS salmon containing no Provian NDV. Growth inhibition by Provian NDV was less prominent at 8 °C storage with 1.2–1.4 log lower *L. monocytogenes* levels for CS salmon in treatments no. 16-19 compared to salmon without Provian NDV (treatments no. 1-2), but with no differences in *L. monocytogenes* levels in CS salmon with 0.5% or 0.9% Provian NDV after 29 days of storage (Table 1, Figure 4).

**Figure 5:**
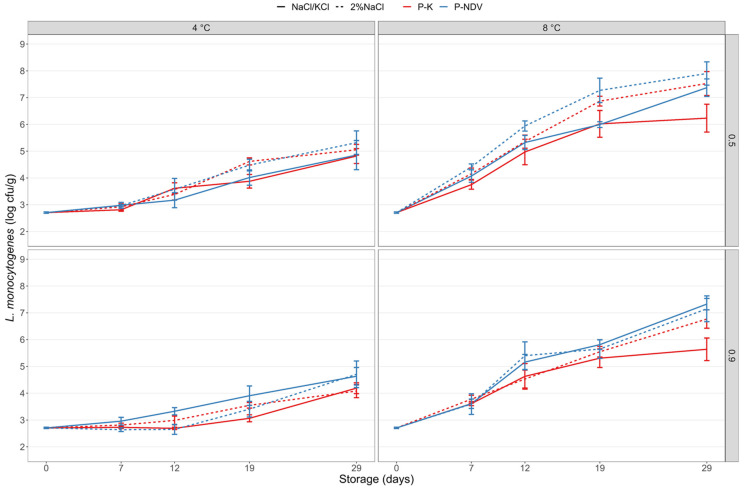
Growth of *L. monocytogenes* in CS-salmon added 3.0% (2.4% NaCl + 0.6% KCl) and 2.5% (2.0% NaCl + 0.5% KCl) mineral salts and Provian K or Provian NDV at levels of 0.5% and 0.9% in the salting process. The CS salmon were inoculated at Day 0 and stored under vacuum at 4 °C and 8 °C for 29 days. Mean values of two experiments (four to eight measurements per data point) and standard error of the mean are displayed.

A certain level of salt is needed to produce CS salmon with essential technological, sensory, and quality characteristics. An effective sodium reduction strategy could be to lower the total amounts of mineral salts and concomitantly partly substitute the NaCl with KCl. CS salmon with reduction in total mineral salt levels from 3% (2.4% NaCl + 0.6% KCl) to 2.5% (2.0% NaCl + 0.5% KCl) and with equal levels of Provian K or Provian NDV demonstrated overall similar growth of *L. monocytogenes* during storage (Figure 5). However, at abuse storage temperature (8 °C) CS salmon with low levels (2.5%) of mineral salts and Provian salts (0.5%) indicated up to 1.3 log higher *L. monocytogenes* counts at end of storage (Day 29) than the CS salmon with 3% mineral salt and the same type and level of Provian salts added. Increasing the Provian salt levels to 0.9% appeared to reduce the *L. monocytogenes* cell count differences under these storage conditions and with somewhat higher growth-inhibiting effects using Provian K compared with Provian NDV (Figure 5).

### 3.4. Physiochemical Characteristics of Salmon with Sodium-Reduced Salt Replacers and Organic Acid Salt Preservatives

No major differences were found in the pH (range 6.21–6.28) and a_w_ (range 0.968–0.973) of CS salmon produced with NaCl or sodium-reduced salt replacers (Treatments no. 1–4, Table 1). The Provian salts did not provide any significant change in pH, giving values in the range 6.20–6.25 and 6.24–6.33 for CS salmon containing Provian K (Treatment no. 10–15) and Provian NDV (Treatment no. 16–21), respectively. Neither different types and levels of Provian salts had significant effects on a_w_ levels (range 0.960–0.972). The effects of the different salt combinations and levels on weight yield of the fillets after salting (98.8 ± 1.4%) and after smoking (96.8 ± 0.2%) were also small.

Levels and uptake of total mineral salts (NaCl + KCl) demonstrated certain variations between the individual samples. The overall average mineral salt uptake was 91% and with no apparent differences in the average uptake in CS salmon containing mineral salts only (Treatment no. 1 through 4) and in types also added Provian salts (Treatments no. 10 through 21).

Analyses demonstrated acetate levels in CS salmon with Provian salts to be in the range of 0.2–0.5%. The acetate levels correlated with levels of Provian salts added. Measured acetate levels were 0.2–0.3% and 0.4–0.5% in samples with added 0.5% and 0.9% Provian K, respectively. For Provian NDV, the corresponding measured levels of acetate were 0.2–0.3% and 0.3–0.5%. This corresponded to an average uptake of 75% of the added acetate in CS salmon. The levels of naturally occurring lactate (range 0.75–0.8%) did not differ substantially in the different CS salmon products (not displayed). 

### 3.5. Sensory and Chemical Properties of Industrially Produced CS Salmon with Salt Replacers and Acetate Containing Preservative Salts

**Figure 6:**
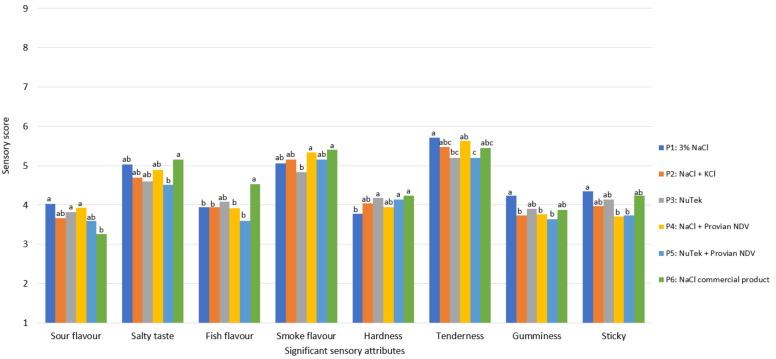
Sensory attributes indicating statistically significant differences after descriptive analysis of CS salmon produced with NaCl and added KCl and inhibiting salt proviant NDV (Products P1–P6). Letters above the columns indicate groupings according to the TUKEY’s HSD test at significance level *p* < 0.05. Samples with the same letter are considered being equal for the specific sensory attribute.

Industrially produced CS salmon with sodium-reduced salt replacers and the organic acid-based preservative salt Provian NDV were evaluated for sensory differences. The salt mixes were prepared and added to obtain the same level of total mineral salts in the CS salmon but with 30% (*w*/*w*) replacement of NaCl by KCl in certain samples and with Provian NDV added to salt mixes with NaCl and a sodium-reduced mix (Product no. P1-P6; Table 2). The descriptive analysis demonstrated only small differences in sensory characteristics between the different CS salmon product types (Table S3). Eight attributes (sour flavor, salty taste, fish flavor, smoke flavor, tenderness, hardness, gumminess, and stickiness) of 23 evaluated sensory properties demonstrated small but statistically significantly different scores between the six products P1-P6 (Figure 6). 

There were no significant differences in pH (range 6.1–6.3) and a_w_ levels (range 0.967–0.979) between sodium-reduced or reference samples produced with NaCl.

### 3.6. Growth of L. monocytogenes in Industrially Produced CS Salmon

**Figure 7:**
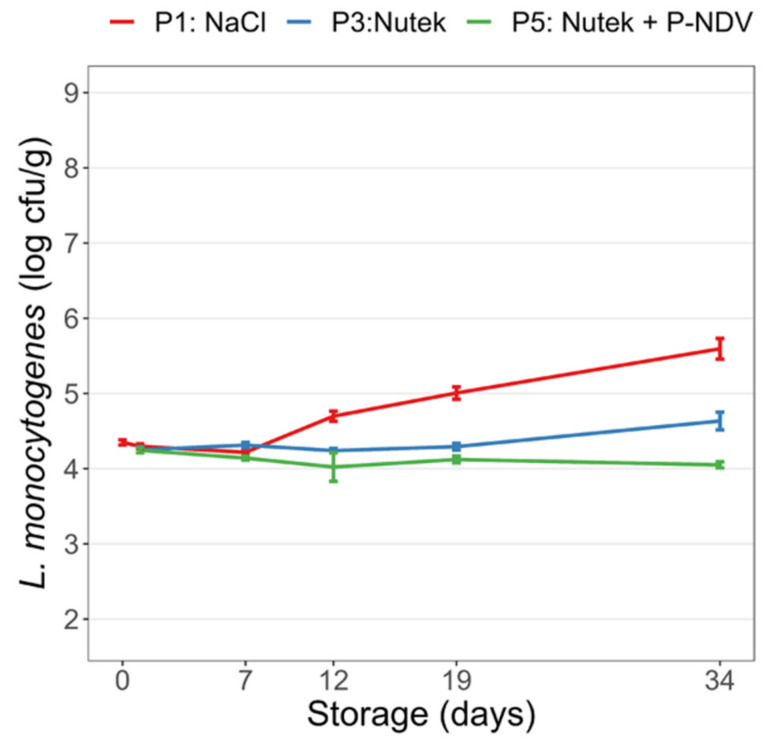
Growth of *L. monocytogenes* in industrially produced CS salmon with NaCl, sodium reduced salt (Nutek), and combined Nutek and Provian NDV preservative salt (products P1, P3, P5, respectively; Table 2). The CS salmon were inoculated at Day 0 and stored under vacuum at 4 °C for 34 days. Mean values of six analysed samples and standard errors of the mean are displayed.

The challenge test performed with industrially produced CS salmon added NaCl (Product P1; Table 2), the salt replacer Nutek (Product P3), or the combination of Nutek and the preservative salt Provian NDV (Product P5) demonstrated limited although statistically significant differences in *L. monocytogenes* growth between the products during 34 days of storage at 4 °C (Figure 7). In CS salmon produced with 3% NaCl, a 1.3 log increase in *L. monocytogenes* levels was observed compared to only a 0.3 log increase observed for CS salmon produced with Nutek during the storage period. No *L. monocytogenes* growth was evident in the CS salmon containing Provian NDV. 

### 3.7. Effects of Sodium-Reduced Salt Replacers on the Indigenous Microbiota 

**Figure 8:**
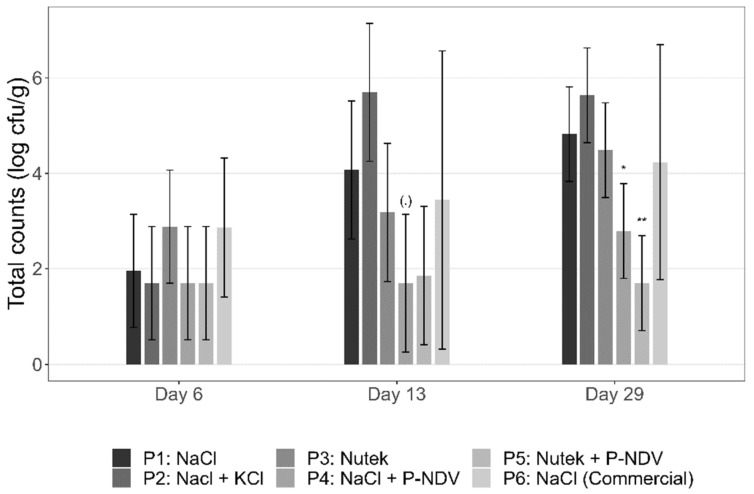
Total viable counts in industrially produced CS salmon with NaCl, sodium-reduced salts and variants with added Provian NDV preservative salt (products P1 through P6; Table 2). Expected means and 95% confidence intervals are displayed for P1-P6. Significant differences to the reference (P1) for each day are displayed with symbols (.) (*p* < 0.1), * (*p* < 0.05) and ** (*p* < 0.01).

**Figure 9:**
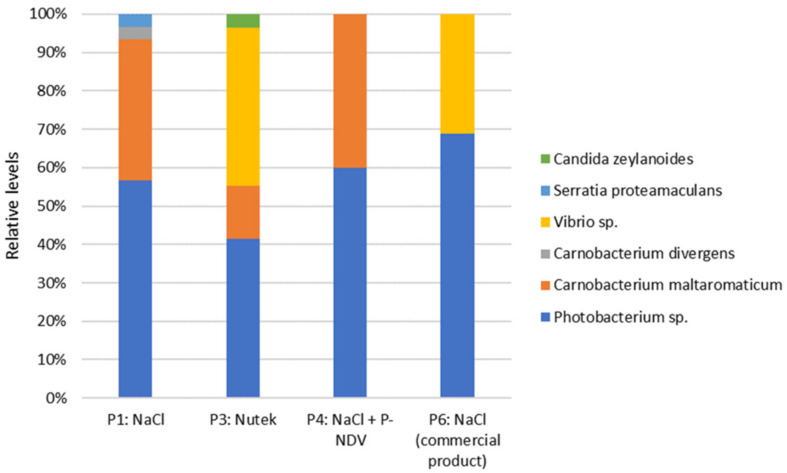
Relative levels of bacteria (and one yeast) in industrially produced CS salmon with NaCl, sodium-reduced salt (Nutek), and variants with added Provian NDV preservative salt. Data obtained by MALDI-TOF MS identification of 30 colonies isolated after 29 days of storage from each sample.

Total viable counts demonstrated generally similar levels (up to 2.9 log/g) in all products P1 through P6 at Day 6 after production (Table 2; Figure 8). Total viable counts in samples with the preservative salt Provian NDV (product P4, P5) remained low (<log 3) during the entire storage period and with significantly lower counts during storage compared to the reference product P1. In samples without Provian NDV, bacterial growth occurred, but remained below log 6 for all product types and with no significant differences between sodium-reduced (product P2, P3) and reference samples (product P1, P6; Figure 8). Microbiota analyses performed by MALDI-TOF MS on isolated colonies of selected samples after 29 days of storage at 4 °C (product P1, P3, P4, P6) demonstrated an overall dominance of *Photobacterium* spp. followed *by Carnobacterium maltaromaticum* and *Vibrio* spp. (Figure 9). Product P5 was excluded from analysis due to low level total viable counts.

## 4. Discussion

To stimulate the salmon industry to produce CS salmon with reduced sodium content, thorough evaluations of the effects of sodium salt reductions or sodium salt substitution on product quality and safety are needed. From the manufacturer’s perspective, a key challenge when replacing NaCl with salt substitutes includes the effects on sensory parameters. Another is the effect of changes in salt types and levels on the food safety risks of CS salmon, particularly with respect to *L. monocytogenes* growth, while maintaining other product quality parameters. Organic acid-based preservative salts including “label-friendly” products with bacterial growth-inhibiting effects have attained attention as a promising approach for enhancing microbial safety and quality in potential risk foods like CS salmon [26,44,45]. 

Several commercially available mineral salts with partial replacement of NaCl with KCl exist. In the present study, salt mixes and commercial sodium salt substitutes with NaCl replaced by KCl (20–50% substitution) were selected as they represent affordable mineral salts with similar technological and functional characteristics as NaCl, and with KCl levels regarded as appropriate in such products [18]. 

The sensory screening revealed generally small differences in taste parameters between CS salmon containing different salt replacers and levels of KCl. High potassium levels have been associated with off-tastes like bitterness and a pungent sensation. However, replacement of 20–40% of NaCl by KCl have been reported to have little effect on the sensory properties of CS salmon or trout [12,46,47]. The present study supports these findings, though also indicates that types and levels of other mineral salts in sodium-reduced salt replacers may affect the sensory properties and thus their suitability for use in CS salmon. Product- and process-specific evaluations therefore are needed to select appropriate commercial sodium-reduced mineral salts that maintain key quality characteristics of CS salmon products, as also indicated by others [19,48].

Only minor differences in *L. monocytogenes* growth were evident between pilot scale-produced CS salmon with NaCl and CS salmon with KCl-based salt substitutes (Table 1, Treatment no. 1–4; Figure 2). All salt types provided highly similar effects on lag time, growth rate, and maximum levels of *L. monocytogenes*. Although CS salmon with Smart Salt 40 demonstrated slightly lower *L. monocytogenes* counts during storage, type of salt explained <1% of the variations on the *L. monocytogenes* levels during storage and therefore appears to have no practical relevance for *L. monocytogenes* levels in the products. No significant effects of salt substitution on a_w_ and pH were observed. The results were as expected according to previous studies, reporting no differences in the bacteriostatic or bactericidal effects imposed by the different cations of Na^+^ or K^+^ and similar antimicrobial activity of NaCl and KCl at equivalent a_w_ [25,49]. Likewise, no pH and a_w_ effects were reported in similar studies [11,19,47]. Storage temperature and storage time explained >90% of the variation in *L. monocytogenes* levels during storage in both salmon produced with NaCl and in sodium-substituted products (Table 1, Treatments no. 1–4; Supplementary Table S2 (ANOVA data). This emphasizes the importance of appropriate cold storage conditions to reduce the *Listeria* food safety risks of such products. 

The present study confirmed that the 2.5–3% mineral salt levels typical for CS salmon did not provide significant growth inhibition of the salt-tolerant *L. monocytogenes*. The inclusion of the acetate-rich salts Provian K and Provian NDV provided clear growth-inhibiting effects of *L. monocytogenes* in both CS salmon with regular NaCl in sodium-replaced and mineral salt-reduced samples. We have recently reported similar effects of the “clean-label” acetate-rich fermentate Verdad N6 applied on CS salmon [26]. The salt’s inhibitory effect is due to undissociated acetic acid that penetrates the bacterial membrane and acidifies the interior of the cell. The Provian salts contain potassium cations and thereby do not contribute sodium ions to the products. For Provian K, growth inhibition was overall dose-dependent with increased inhibition obtained with higher levels of added Provian K (Figure 3). The results indicated that Provian K provided slightly decreased growth-inhibition effects in CS salmon with regular NaCl compared with sodium-reduced samples. However, statistically significant differences were not consistent and only observed at certain time points, storage temperatures and concentrations of the Provian salt (Supplementary Table S2) The dose-dependent effect of Provian NDV was evident for CS salmon with 3% NaCl, but less clear for sodium-reduced salmon where higher added levels (0.9%) did not always provide significantly lower *L. monocytogenes* levels compared with added low level (0.5%) Provian NDV (Figure 4). The differences in measured acetate levels (up to 0.2 percentage points) between CS salmon with added 0.5% and 0.9% Provian salts reflect the variable dose-dependent effects generally obtained. 

Another potential sodium reduction strategy could be reductions in the total levels of mineral salts while including preservative organic acid salt ingredients to ensure high quality and microbiologically safe products. In this case, the inhibition of *L. monocytogenes* remained similar in samples with both 3% and 2.5% total mineral salt, except for the higher growth in samples with 2.5% total mineral salt and 0.5% preservative salt at abuse temperature of 8 °C (Figure 5). These findings illustrate the importance of using salt levels and salting procedures that ensure sufficient levels of mineral (and preservative) salts in the products and that CS salmon with reduced total salt, from a microbial food safety perspective, may be somewhat less robust when exposed to abuse temperatures (e.g., 8 °C vs. 4 °C) and the prolonged storage typical for CS salmon products. 

Salmon is a non-homogenous raw material, and variations in content of fat, water, and other substances like sugar and minerals, in addition to extensive variations in CS salmon processing factors, affect the physiochemical properties of the final products. During processing, variations in exposure to salts and antimicrobial smoke components may occur between and within fillets. Thinner parts of the fillets may obtain a higher salt concentration, increased levels of phenols, and a more extensive drying. This may partly explain the observed variations in the levels and uptake of mineral salts and acetate (from the preservative Provian salts) in the current study. Large variations in sodium content of CS salmon reference products with added NaCl and in samples with added salt substitutes were recently reported and explained by variations in fat content of the salmon and the salting procedure [11]. We found the uptake of mineral salts also depends on the salting procedure (salting in the pilot plant vs. salting in the commercial processing plant) and not on salt type or the presence or absence of Provian salts. Although not directly comparable, lower uptake of the added salts was evident in the salting procedure applied in-industry compared with the controlled in-pilot plant salting performed in plastic bags. Controlled in-pilot plant salting still provided relatively high standard errors on measured salt. Several reports have described significant variations in pH, a_w_, and phenol content in the CS salmon under study. The in-pilot-plant-produced CS salmon of the current study (Treatments no. 1 through 21) was in the higher pH (6.20–6.33) and a_w_ ranges (0.960–0.973), and lower phenol concentration range (3.4–4.8 ppm) compared with ranges of similar studies [11,19,26,39,50,51,52]. These variations in CS salmon parameters reflect not only non-uniform processing conditions including salting and smoking procedures, but also variations in physiochemical parameters of the salmon raw materials. For the industry-produced salmon, about 30% of the added acetate (from Provian NDV) was absorbed, giving a final acetate level in the CS salmon of 0.2%. This led to effective growth inhibition of *L. monocytogenes*. Similar acetate levels were predicted to ensure growth inhibition of *L. monocytogenes* in a salt-substituted CS salmon product, although a lower pH and a_w_ compared to our study further contributed to the growth inhibitory effects on *L. monocytogenes* [19]. We observed limited growth (1.2 log) in the industrially produced control CS salmon with 3% NaCl. In the sodium-reduced salmon, growth was even lower. The reason for this low growth is still an open question, but this could indicate a presence of other factors restricting substantial *L. monocytogenes* growth in the products. We have previously demonstrated that increased smoking of the fillets can significantly reduce the growth of *L. monocytogenes* [26], and fillets may receive different amounts of growth-inhibiting smoke depending on the position in the smoking chamber. Other studies have documented that small changes in preserving parameters (e.g., pH and salt levels) have effects on *L. monocytogenes* growth [38,53], although variations in pH and a_w_ of the industrially produced CS salmon could probably not explain the observed growth effects on *L. monocytogenes* in the present study. 

Background microbiota can also inhibit *L. monocytogenes* growth. Total counts were below 3 log cfu/g six days after production and did not exceed 6 log cfu/g after 29 days storage at 4 °C, and it appears unlikely that the background microbiota is responsible for the low *L. monocytogenes* levels observed. The results also indicated effective growth inhibition of the background microbiota in CS salmon with Provian NDV. The microbiota was dominated by *Photobacterium* followed by *Carnobacterium*. Others have also demonstrated *Photobacterium* and lactic acid bacteria to be among the dominating bacteria in vacuum-packed CS salmon [54,55,56,57,58]. Due to the experimental setup and the relatively low acetate levels in the CS salmon in the current study, the data could not confirm our previous results indicating reduced relative levels of *Photobacterium* and increased relative levels of *Carnobacterium* in CS salmon with increasing levels of acetate [26]. 

Growth of *L. monocytogenes* in CS salmon was generally independent of the type of mineral salt, but the paramount importance of keeping a continuous cold chain from production through storage and to consumption was underscored. The organic acid salts Provian K and Provian NDV generally demonstrated similar and substantial growth-inhibiting effects of *L. monocytogenes* independent of the type of mineral salt used. Growth inhibition of the CS salmon background microbiota was also observed and indicates that such mitigation strategies might be effective and useful to produce microbiologically safe and high-quality CS salmon with a prolonged shelf life. A potential challenge for cost-effective and confident use of such preventive salts could be uneven and limited uptake and distribution of added salts in salmon fillets. This could lead to non-uniform products with uneven and too-low levels of salt in the products, having potential impact for CS salmon quality and safety.

In conclusion, the present study has demonstrated that sodium reduction and improved microbial food safety of CS salmon appear to be feasible through combined use of sodium-reduced commercially available mineral salts and preservative salts without compromising key sensory and quality parameters. Replacement of 20–40% of NaCl with KCl and added levels of 0.5–0.9% acetate-based preservative salts appears adequate to obtain healthier and microbiologically safer CS salmon products. However, thorough process-specific evaluation and standardization of the salting process are warranted to achieve sufficient uptake of salts and ensure high-quality product characteristics according to safety, sensory, and consumer preference of such products. 

## Figures and Tables

**Table 1:** Characteristics of produced CS-salmon applied in the sensory screening (S) and/or *L. monocytogenes* challenge tests (L).

Treatment No.	Salt Mixture ^1^	Experiments Where Applied	Added NaCl, KCl, Total Mineral Salts (%) ^2^			Proportion of KCl and Other Non-NaCl Mineral Salts in Mix	Added Organic Acid Salt (%) ^2^	Measured Product Characteristics				
			NaCl	KCl	Total			Total Mineral Salts (% of Fish) ^3^	Total Mineral Salts Uptake (%) ^4^	Acetate (%)	Acetate Uptake (%) ^5^	Phenol (ppm)
1	NaCl (control)	S; L	3.0	-	3.0	-	-	2.2 ± 0.1	72	-	-	3.66
2	NaCl + KCl	S; L	2.4	0.6	3.0	20% KCl	-	2.5 ± 0.2	86	-	-	-
3	Nutek 78300	S; L	2.1	0.9	3.0	30% KCl	-	2.9 ± 0.6	105	-	-	-
4	Smart Salt	S; L	1.8	0.64	3.0	21.4% KCl; 15.5% MgCl_2_	-	2.9 ± 0.6	101	-	-	-
5	FlakeSelect	S	1.5	1.5	3.0	50% KCl;	-	-	-	-	-	-
6	Lomasalt 2.0	S	1.5	0.84	3.0	30% KCl; 22 % MgCO_3_	-	-	-	-	-	-
7	Nutek 15000	S	2.1	0.9	3.0	30% KCl	-	-	-	-	-	-
8	Saltwell Reg.	S	1.95	0.9	2.9	30% KCl	-	-	-	-	-	-
9	SOLO-LITE	S	2.1	0.87	3.0	30% KCl; 1% MgSO_4_	-	-	-	-	-	-
10	NaCl + P-K	L	3.0			-	0.5	2.1 ± 0.4	71	0.2 ± 0.0	77	-
11	NaCl + P-K	L	3.0			-	0.9	1.9 ± 0.7	68	0.4 ± 0.0	68	-
12	NaCl + KCl + P-K	L	2.4	0.6	3.0	20% KCl	0.5	2.6 ± 0.2	92	0.3 ± 0.1	89	-
13	NaCl + KCl + P-K	L	2.4	0.6	3.0	20% KCl	0.9	2.5 ± 0.3	89	0.5 ± 0.0	79	-
14	NaCl + KCl + P-K	L	2.0	0.5	2.5	20% KCl	0.5	2.3 ± 0.3	96	0.2 ± 0.0	50	-
15	NaCl + KCl + P-K	L	2.0	0.5	2.5	20% KCl	0.9	2.0 ± 0.2	85	0.4 ± 0.1	76	-
16	NaCl + P-NDV	L	3.0	-	3.0	-	0.5	2.6 ± 0.4	85	0.2 ± 0.1	72	4.78
17	NaCl + P-NDV	L	3.0	-	3.0	-	0.9	2.7 ± 0.2	90	0.3 ± 0.1	61	-
18	NaCl + KCl + P-NDV	L	2.4	0.6	3.0	20% KCl	0.5	3.1 ± 0.3	104	0.2 ± 0.0	71	-
19	NaCl + KCl + P-NDV	L	2.4	0.6	3.0	20% KCl	0.9	2.7 ± 0.2	94	0.3 ± 0.0	57	-
20	NaCl + KCl + P-NDV	L	2.0	0.5	2.5	20% KCl	0.5	3.3 ± 0.7	134	0.4 ± 0.1	119	-
21	NaCl + KCl + P-NDV	L	2.0	0.5	2.5	20% KCl	0.9	2.2 ± 0.2	91	0.5 ± 0.1	82	4.19

^1^ P-K: Provian K, P-NDV: Provian NDV; ^2^ Added relative to the weight of the salmon fillet; ^3^ Total Cl^−^ was measured and values calculated according to the relative amounts of different mineral salts; ^4^ Uptake of mineral salts, expressed as percentage of total added; ^5^ Uptake of acetate expressed as percentage of acetate from added Provian salts (Provian K: 20% K-diacetate, 80% K-acetate; Provian NDV: 100% K-acetate).

**Table 3:** *Listeria monocytogenes* strains.

Strain No.	Serotype	MLVA/ST ^1^	Source ^2^	Other Designations; Reference
MF3860	1/2 a	6-10-5-16-6/20	Salmon processing, Plant S4	[32]
MF3939	1/2 a	5-8-15-10-6/14	Salmon processing, Plant S3	[32]
MF4001	1/2 a	5-8-15-10-6/14	Salmon processing, Plant S2	[32]
MF4077	1/2 a	6-9-18-16-6/8	Salmon processing, Plant S1	[32]
MF4588	1/2 a	7-7-10-10-6/7	Salmon processing, Plant S1	[32]
MF4804	1/2 a	6-7-14-10-6/121	Salmon processing, Plant S2	[32]
MF2184	1/2 b	7-8-0-16-0/3	Meat processing, outbreak	2583/92; [33]
MF3009	1/2 b	n.d./5	Cattle	FSL J2-064; [34,35]
MF3039	4 b	n.d./6	Human, cerebrospinal fluid, outbreak	FSL N1-227; [34,35]
MF3710	4 b	7-7-20-6-10/n.d.	Human, cerebrospinal fluid	CCUG3998; Culture Collection University of Gothenburg

^1^ MLVA designation according to Møretrø et al. [32]. ST numbers refer to Institute Pasteur MLST database (http://bigsdb.web.pasteur.fr./listeria/listeria.html; accessed on 23 April 2022). ^2^ Plant designation according to Møretrø et al. [32].

## Data Availability

All related data and methods are presented in this paper. Additional inquiries should be addressed to the corresponding author.

## Supplementary Materials

The following supporting information can be downloaded at: https://www.mdpi.com/article/10.3390/foods11101483/s1, Table S1: Sensory attributes and their descriptions used in the sensory descriptive analysis of industrially produced CS salmon with salt replacers; Table S2: Anova data; Table S3: Sensory intensity scores of 23 sensory attributes related to smell, taste and texture of industrially produced CS salmon.
