# Supplementary material for: Microbial Safety and Sensory Analyses of Cold-Smoked Salmon Produced with Sodium-Reduced Mineral Salts and Organic Acid Salts
Source: Foods. 2022 May 19;11(10):1483. doi: 10.3390/foods11101483 (PMC9141012; doi:10.3390/foods11101483)
Supplement: Supplementary file 1 [file foods-11-01483-s001.zip › Table S1 .pdf]

Table S1. Sensory attributes and their descriptions used in the sensory descriptive analysis of industrially produced CS salmon with salt replacers.

**Attribute**	**Description**
Smells	
Sour smell	Organoleptic attribute of pure substances or mixtures which produces the sour sensation
Fish smell	Relates to the smell of fish
Smoke smell	Describes the smell of smoke, campfire, or smoke condensate
Cloying smell	Describes an unfresh/sickening smell
Pungent	Describes a product causing a sharp sensation of the buccal and nasal mucous membranes.
Rancid smell	The intensity of all rancid smells (i.e. gras, hay, stearin, paint)
Total smell intensity	Describes the total intensity of smells
Tastes and flavours	
Sour flavour	Organoleptic attribute of pure substances or mixtures which produces the sour sensation
Salty taste	Describes the basic taste produced by diluted aqueous solutions of various substances such as sodium chloride
Bitter taste	Describes the basic taste produced by diluted aqueous solutions of various substances such as quinine and caffeine
Umami taste	Describes the basic taste of umami
Fish flavour	Relates to the flavour of raw fish
Smoke flavour	Describes the flavour of smoke, campfire, and smoke condensate
Metal taste	Describes the taste of metal (ferrous sulphate)
Cloying flavour	Describes an unfresh/sickening taste/flavour
Rancid flavour	Describes the intensity of all rancid flavours (i.e. gras, hay, stearin, paint)
Texture	
Hardness	Describes a mechanical texture attribute related to the force needed to bite through the sample. Evaluated by the first bite
Juiciness	Describes a surface textural attribute that describes liquid emitted from a product. Fluid released from the sample after 4-5 chews
Tenderness	Describes a mechanical texture attribute related to time and number of chews necessary to swallow the sample
Gumminess	Describes the mechanical texture attribute related to the cohesiveness of a tender product (i.e. gummy)
Sticky	Describes the mechanical texture attribute related to the force required to remove material that adheres to the mouth
Astringency	Describes the organoleptic attribute of pure substances or mixtures which produces the astringent sensation

After taste	The olfactory and/or gustatory sensation which occurs after the elimination of the product, and which differs from the sensation perceived whilst the product was in the mouth
