# Supplementary material for: Microbial Safety and Sensory Analyses of Cold-Smoked Salmon Produced with Sodium-Reduced Mineral Salts and Organic Acid Salts
Source: Foods. 2022 May 19;11(10):1483. doi: 10.3390/foods11101483 (PMC9141012; doi:10.3390/foods11101483)
Supplement: Supplementary file 1 [file foods-11-01483-s001.zip › Table S3.pdf]

**Table S3.** Sensory intensity scores of 23 sensory attributes related to smell, taste and texture of industrially produced CS salmon (Product no. P1-P6). Products associated with the same letters are not significantly different at 5% level.

| | **P1_3% NaCl** | **P2_NaCl + KCl** | **P3_Nutek** | **P4_NaCl + Provian NDV** | **P5_Nutek + Provian NDV** | **P6_NaCl commercial product** | ***P-value*** |
|---|---|---|---|---|---|---|---|
| Sour smell | 4.29 A | 4.24 A | 3.95 A | 4.24 A | 4.11 A | 3.53 A | *0.075* |
| Fish smell | 3.71 A | 3.78 A | 3.74 A | 3.76 A | 3.62 A | 4.09 A | *0.911* |
| Smoke smell | 5.04 A | 5.13 A | 4.82 A | 5.14 A | 5.23 A | 5.37 A | *0.052* |
| Cloying smell | 1.94 A | 1.87 A | 1.96 A | 1.91 A | 1.98 A | 2.63 A | *0.278* |
| Pungent | 1.27 A | 1.23 A | 1.31 A | 2.06 A | 1.97 A | 1.48 A | *0.012* |
| Rancid smell | 1.53 A | 1.46 A | 1.47 A | 1.43 A | 1.44 A | 1.90 A | *0.200* |
| Total smell intensity | 5.21 A | 5.16 A | 5.09 A | 5.27 A | 5.26 A | 5.38 A | *0.311* |
| Sour flavour | 4.02 A | 3.66 AB | 3.82 A | 3.93 A | 3.60 AB | 3.26 B | ***0.016*** |
| Salty taste | 5.03 AB | 4.70 AB | 4.60 AB | 4.89 AB | 4.51 B | 5.15 A | ***0.024*** |
| Bitter taste | 3.96 A | 4.09 A | 4.05 A | 3.94 A | 4.13 A | 4.34 A | *0.585* |
| Umami taste | 3.74 A | 3.42 A | 3.46 A | 3.62 A | 3.40 A | 3.40 A | *0.243* |
| Fish flavour | 3.95 B | 3.94 B | 4.08 AB | 3.91 B | 3.60 B | 4.53 A | ***0.036*** |
| Smoke flavour | 5.06 AB | 5.15 AB | 4.84 B | 5.34 A | 5.16 AB | 5.41 A | ***0.016*** |
| Metal taste | 3.71 B | 3.88 AB | 3.89 AB | 3.64 B | 3.73 B | 4.19 A | *0.249* |
| Cloying flavour | 2.01 A | 2.08 A | 2.15 A | 1.94 A | 2.39 A | 2.44 A | *0.516* |
| Rancid flavour | 1.55 A | 1.61 A | 1.43 A | 1.60 A | 1.47 A | 1.95 A | *0.099* |
| Hardness | 3.78 B | 4.04 AB | 4.18 A | 3.95 AB | 4.14 A | 4.24 A | ***0.010*** |
| Juiciness | 4.69 A | 4.67 A | 4.71 A | 4.80 A | 4.60 A | 4.56 A | *0.559* |
| Tenderness | 5.71 A | 5.48 ABC | 5.20 BC | 5.63 AB | 5.19 C | 5.45 ABC | ***0.003*** |
| Gumminess | 4.23 A | 3.74 B | 3.90 AB | 3.76 B | 3.63 B | 3.88 AB | ***0.004*** |
| Sticky | 4.35 A | 3.97 AB | 4.14 AB | 3.71 B | 3.74 B | 4.23 AB | ***0.008*** |
| Astringency | 3.75 A | 3.72 A | 3.65 A | 3.68 A | 3.84 A | 3.93 A | *0.625* |
| After taste | 5.46 A | 5.62 A | 5.45 A | 5.59 A | 5.70 A | 5.92 A | *0.229* |
