# Supplementary material for: Microbial Safety and Sensory Analyses of Cold-Smoked Salmon Produced with Sodium-Reduced Mineral Salts and Organic Acid Salts
Source: Foods. 2022 May 19;11(10):1483. doi: 10.3390/foods11101483 (PMC9141012; doi:10.3390/foods11101483)
Supplement: Supplementary file 1 [file foods-11-01483-s001.zip › Tables S2 ANOVA data.pdf]

Supplemental ANOVA data Table S2 - ANOVA tables

Five different analyses were performed, to test

i) Effect of different mineral salts

ii) Effect of Provian K

iii) Effect of Provian NDV

iv) Comparison of Provian K and Provian NDV

v) Effect of Provian K/NDV with salt reduction

Model selection and testing

For all ANOVA models applied, type III tests are performed due to imbalance as there are more observations on control samples than other treatments. For complex models' backward elimination using the AIC criterium (r-function step) was applied to reduce the model. In all cases where model selection was performed, we checked that the reduced model was not significantly different from the full model.

For all models the dependent variable is the log growth of *L. monocytogenes.* Days of storage (Day, 0, 7, 12, 19 and 29) and temperature (Temp, 4°C or 8°C) and their interaction is included in all models. The two-way model comprising Day, Temp and their interaction is referred to as the null model.

- Salt (different levels for the different analyses)
    i. 3.0% NaCl or 2.4% NaCl+0.6% KCl
    ii. 3.0% NaCl or 2.4% NaCl+0.6% KCl
    iii. 3.0% NaCl or 2.4% NaCl+0.6% KCl
    iv. 2.0% NaCl+0.5% KCL or 2.4% NaCl+0.6% KCl
    v. 2.0% NaCl + 0.5% KCl or 2.4% NaCl+0.6% KCl
- Provian K (0, 0.5 % and 0.9 %) – part ii + v
- Provian NDV (0, 0.5 % and 0.9 %) – part iii + v
- Concentration of Provian (K or NDV) (0.5% or 0.9%) – part iv
- Salt reduction with salt as 2.4% NaCl+0.6% KCl, 2.0% NaCl (part v)
- e: random error

All models are compared to a null model, describing the growth of L. monocytogenes using day and temperature only, according to

$$y \sim Day + Temp + Day{:}Temp + e \qquad \text{(S1)}$$

The colon indicates an interaction effect.

i) Effect of different mineral salts

Factors included in the modelling in addition to day and temperature are

- 3.0% NaCl (control),
- 2.4% NaCl+0.6% KCl
- Nutek
- Smart Salt

The model was only nearly significant compared to the null model (Eq. S1, p-value 0.09). Table ANOVA S2a shows the ANOVA for the full model.

Table ANOVA S2a: *ANOVA (type III) for analysis i), effect of different mineral salts on growth of L. monocytogenes.*

	Sum Sq	Df	F value	Pr(>F)	Explained Variance
(Intercept)	6066.349	1	42612.213	0.000	
Salt	2.865	3	6.709	0.000	0.656
Temp	130.706	1	918.128	0.000	29.927
Day	262.437	3	614.483	0.000	60.089
Salt:Temp	0.081	3	0.189	0.904	0.018
Salt:Day	0.362	9	0.282	0.979	0.083
Temp:Day	20.796	3	48.692	0.000	4.761
Residuals	19.504	137			4.466

ii) Effect of Provian K

Factors included in the model in addition to day and temperature are

- Provian K (0, 0.5% or 0.9%)
- Salt (3.0% NaCl or 2.4% NaCl+0.6% KCl)

Table ANOVA S2b: *ANOVA (type III) for analysis ii), effect of Provian K for different salts and concentrations on growth of L. monocytogenes.*

	Sum Sq	Df	F value	Pr(>F)	Explained Variance
(Intercept)	5409.407	1	19617.614	0.000	
Provian K	232.394	2	421.398	0.000	32.936
Salt	4.140	1	15.013	0.000	0.587
Temp	164.870	1	597.913	0.000	23.366
Day	207.481	3	250.815	0.000	29.405
Provian K:Salt	1.862	2	3.376	0.036	0.264
Provian K:Temp	0.468	2	0.849	0.429	0.066
Provian K:Day	17.407	6	10.521	0.000	2.467
Salt:Temp	0.586	1	2.124	0.147	0.083
Temp:Day	11.223	3	13.567	0.000	1.591
Provian K:Salt:Temp	1.525	2	2.765	0.066	0.216
Provian K:Temp:Day	10.697	6	6.466	0.000	1.516
Residuals	52.943	192			7.503

iii) Effect of Provian NDV

Factors included in the model in addition to day and temperature are

- Provian NDV (denoted Provian in the table, at concentrations 0, 0.5% or 0.9%)
- Salt (3.0% NaCl or 2.4% NaCl+0.6% KCl)

Table ANOVA S2c: *ANOVA (type III) for analysis iii), effect of Provian NDV for different salts and concentrations on growth of L. monocytogenes*

	Sum Sq	Df	F value	Pr(>F)	Explained Variance
(Intercept)	5837.298	1	27339.249	0.000	
Provian	172.976	2	405.070	0.000	24.783
Salt	0.463	1	2.169	0.142	0.066
Temp	188.199	1	881.440	0.000	26.964
Day	253.054	3	395.064	0.000	36.257
Provian:Salt	3.802	2	8.903	0.000	0.545
Provian:Temp	0.186	2	0.435	0.648	0.027
Provian:Day	8.255	6	6.444	0.000	1.183
Temp:Day	8.304	3	12.964	0.000	1.190
Provian:Temp:Day	20.866	6	16.288	0.000	2.990
Residuals	41.849	196			5.996

iv) Comparison of Provian type (K or NDV) at different concentrations

Factors included in the model in addition to day and temperature are

- Provian type (denoted Type, levels and K or NDV)
- Provian concentration (denoted Conc., 0.5% or 0.9%).

Table ANOVA S2d: *ANOVA (type III) for analysis iv), effect of Provian type (K or NDV) for different concentrations of different salts and concentrations on growth of L. monocytogenes*

	Sum Sq	Df	F value	Pr(>F)	Explained Variance
(Intercept)	5261.49	1	13985.73	0.00	
Type	4.58	1	12.18	0.00	0.84
Conc	22.64	1	60.19	0.00	4.17
Temp	205.57	1	546.42	0.00	37.89
Day	200.26	3	177.44	0.00	36.91
Type: Conc	0.92	1	2.46	0.12	0.17
Type:Temp	0.30	1	0.81	0.37	0.06
Type:Day	1.51	3	1.33	0.26	0.28
Temp:Day	15.42	3	13.67	0.00	2.84
Type:Temp:Day	2.94	3	2.60	0.05	0.54
Residuals	88.41	235			16.29

v) Effect of salt reduction and Provian type

Factors included in the model in addition to day and temperature are

- Salt (2.0% NaCl + 0.5% KCl or 2.4% NaCl + 0.6% KCl)
- Provian type (denoted Type; K or NDV) and
- Provian concentration (denoted Conc, 0.5% or 0.9%).

Table ANOVA S2e: *ANOVA (type III) for analysis v), effect of Provian type (K or NDV), Provian concentration in product with reduced salt levels on growth of L. monocytogenes*

	Sum Sq	Df	F value	Pr(>F)	Explained Variance
(Intercept)	5360.478	1	16750.243	0.000	
Type	5.893	1	18.413	0.000	1.027
Conc	19.804	1	61.884	0.000	3.451
Salt	4.489	1	14.027	0.000	0.782
Temp	222.873	1	696.425	0.000	38.841
Day	216.568	3	225.575	0.000	37.742
Type:Conc	0.332	1	1.038	0.309	0.058
Type:Salt	0.751	1	2.345	0.127	0.131
Type:Temp	1.464	1	4.575	0.033	0.255
Type:Day	2.542	3	2.648	0.050	0.443
Conc:Salt	3.212	1	10.038	0.002	0.560
Conc:Day	2.687	3	2.798	0.041	0.468
Salt:Temp	1.920	1	6.000	0.015	0.335
Temp:Day	16.046	3	16.713	0.000	2.796
Type:Conc:Salt	0.982	1	3.070	0.081	0.171
Residuals	74.246	232			12.939
